# Investigating Team Coordination in Baseball Using a Novel Joint Decision Making Paradigm

**DOI:** 10.3389/fpsyg.2017.00907

**Published:** 2017-06-07

**Authors:** Rob Gray, Nancy J. Cooke, Nathan J. McNeese, Jaimie McNabb

**Affiliations:** Human Systems Engineering, Arizona State University Polytechnic, MesaAZ, United States

**Keywords:** teamwork, coordination, cognition, sports, decision making

## Abstract

A novel joint decision making paradigm for assessing team coordination was developed and tested using baseball infielders. Balls launched onto an infield at different trajectories were filmed using four video cameras that were each placed at one of the typical positions of the four infielders. Each participant viewed temporally occluded videos for one of the four positions and were asked to say either “ball” if they would attempt to field it or the name of the bag that they would cover. The evaluation of two experienced coaches was used to assign a group coordination score for each trajectory and group decision times were calculated. Thirty groups of 4 current college baseball players were: (i) teammates (players from same team/view from own position), (ii) non-teammates (players from different teams/view from own position), or (iii) scrambled teammates (players from same team/view not from own position). Teammates performed significantly better (i.e., faster and more coordinated decisions) than the other two groups, whereas scrambled teammates performed significantly better than non-teammates. These findings suggest that team coordination is achieved through both experience with one’s teammates’ responses to particular events (e.g., a ball hit up the middle) and one’s own general action capabilities (e.g., running speed). The sensitivity of our joint decision making paradigm to group makeup provides support for its use as a method for studying team coordination.

## Introduction

Whether executing a “set piece” in soccer, playing a zone defense in football, or turning a double play in baseball, effective performance in team sports hinges on the development of team cognition. Team cognition is the cognitive activity at the team level and is shared amongst team members through interactions in the form of direct or indirect communication and coordination (Cooke et al., 2013). To date, our understanding of team cognition has been limited by the methodologies used to study it which tend to fall into one of two categories (reviewed in McNeese et al., 2016, 2017): (i) knowledge elicitation methods which pool and aggregate the passive responses of individual teammates taken out of context to assess shared mental models (Cannon-Bowers et al., 1993), or (ii) techniques which analyze the macro level behavior of teammates during actual gameplay (e.g., the movements of players from GPS data), but do little to elucidate the underlying perceptual-cognitive processes. The goal of the present study was to develop and test a new paradigm for studying team coordination that represents a middle ground between these two extremes.

**FIGURE 1:**
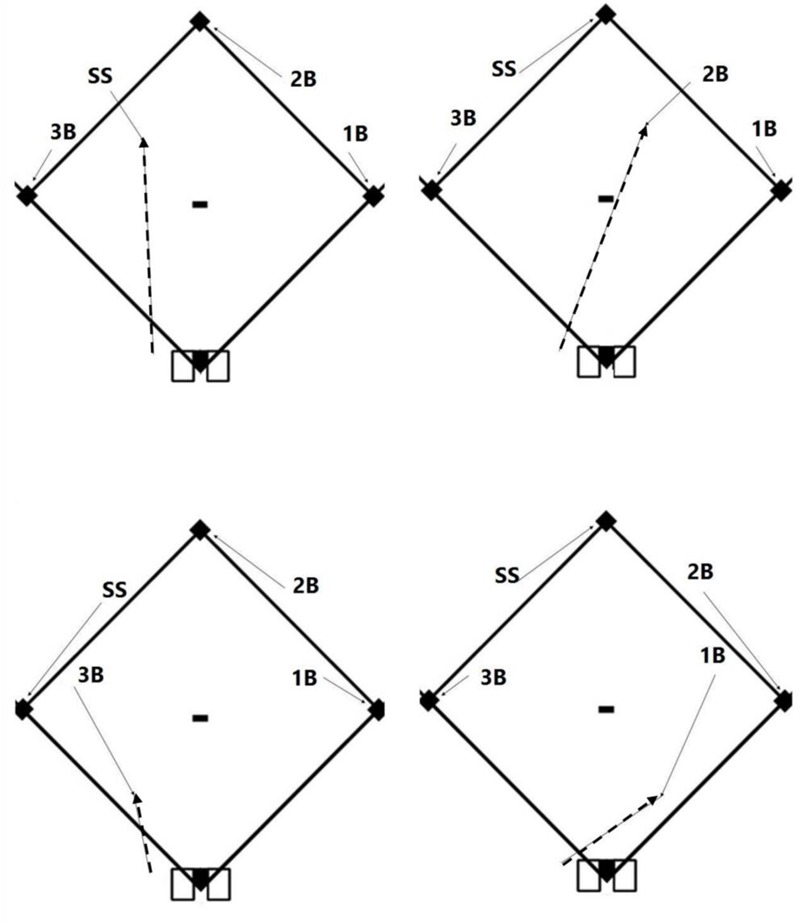
Illustration of coordination in a baseball infield. Depending on the trajectory of the batted ball (dashed lines), each player must decide whether to attempt to move to intercept the ball (i.e., “field it”) or to move so that they can receive a ball thrown to one of the bases (black diamonds). Each of the four panels illustrates how players are typically taught to move (dotted lines) depending on which player fields the ball. 1B = first baseman, 2B = second baseman, SS = shortstop, 3B = third baseman.

A key element of team cognition is coordinated decision making, or as players often refer to: “being on the same page.” An example of the importance of coordinated decision making can be seen on the baseball field. As illustrated in **Figure 1**, when a ball is hit on the ground and there are runners on base, each of the four infielders must rapidly decide between two options: (i) attempting to move to intercept (“field”) the ball, or (ii) moving toward (“covering”) one of bases in preparation to receive a throw. For the two middle infielders (i.e., the shortstop and second baseman) there is further complexity in that they must also decide which base to cover (e.g., a shortstop needs to cover second base if the second baseman fields the ball and third base if the third baseman fields it). In this situation, it is possible to assess the “correctness” of an individual player’s decision — if a player is closest to the ball and decides to field it we could consider it to be a correct decision. However, successfully making an out on the play hinges more on the overall coordination of the teammates’ decisions as opposed to their individual correctness (e.g., if a different player is going to field the ball the overall outcome would be better if the closest player decides to cover a bag). Team decision making in a baseball infield is a prime example of Interactive Team Cognition (ITC) which proposes that team cognition is a dynamic team level activity that is inseparable from the context in which it occurs (Cooke et al., 2013).

As we have proposed previously (McNeese et al., 2016), a fruitful approach for studying team coordination in this context may be to “scale up to a team level” methods that have proven to be effective for assessing perceptual-cognitive processes in individual athletes. For example, the occlusion paradigm has been used to study anticipation and decision making in sports (Abernethy and Russell, 1984). This paradigm involves having an individual view an unfolding event (e.g., an opponent serving a tennis ball) either on video or live, and spatially (e.g., blocking the view of the server’s legs for the entire serve) or temporally (e.g., completely blocking view of the server after 500 ms) occluding the event. Then, asking the viewer to make a decision using either a passive (e.g., saying “down the line or cross court”) or active (e.g., stepping in the anticipated direction of ball travel) response. As reviewed in Farrow and Abernethy (2015), this methodology has been used to understand expertise differences in decision making, anticipation and gaze behavior (at the individual athlete level) for both one-on-one (e.g., tennis or squash serves, soccer penalty kicks) and sporting actions involving multiple players (e.g., deciding whom to pass the ball to in basketball).

In the present study, we extended this occlusion paradigm to create and test a novel method for assessing coordinated decision making in sports. Specifically, a video of an unfolding event (a ball hit onto a baseball infield) was simultaneously filmed from multiple locations, each corresponding to the position of one of the four infielders. Experienced baseball players (in groups of four) were then asked to make coincident decisions (either play the ball or cover a bag) while watching temporally occluded videos of balls hit at different trajectories. The evaluation of two experienced coaches were used to assign a coordination score for each trajectory for each group. In addition, mean decision times were also calculated. Coordination scores ranged from 3-indicating effective coordination (i.e., all bases covered and player identified by the coaches goes for ball) to 0-indicating poor coordination (i.e., no player goes for ball). There were 3 types of groups: (i) *teammates* (players from same team who each viewed videos from the camera location corresponding to their own playing position), (ii) *non-teammates* (players from different teams who viewed videos corresponding to their own position), and (iii) *scrambled teammates* (players from the same team that viewed videos from a camera position that did not correspond to their own playing position). The main goal of the study was validate this new joint decision making paradigm by determining whether or not it is sensitive to group makeup (e.g., teammates vs. non-teammates). Based on the assumption that it would be sensitive, we made the following specific predictions:

(i) The teammate group would have significantly higher coordination scores and significantly faster decision times as compared to the other two groups due to their knowledge of how their teammates act in different game situations and their action capabilities.
(ii) The non-teammates group would have significantly higher coordination scores and significantly faster decision times than the scrambled teammates group because they had more experience playing the viewed position.

## Materials and Methods

### Participants

The participants in the study were 120 male baseball players who played for Division 1 college baseball teams affiliated with the National Junior College Athletic Association (NJCAA, United States) at the time of participation. The mean age of these participants was 20.7 (*SD* = 2.1), the mean number of years of competitive playing experience was 12.2 (*SD* = 1.8), and the mean fielding percentage was 0.92. This study was carried out in accordance with the recommendations of the Arizona State University Institutional Review Board with written informed consent from all subjects. All subjects gave written informed consent in accordance with the Declaration of Helsinki. The protocol was approved by the Arizona State University Institutional Review Board.

The 120 participants were divided into 30 groups of four players, with each group having one first baseman, one second baseman, one shortstop, and one third baseman. There were three group types:

*Teammates* – four players who currently played on the same team together and were asked to make judgments from the viewing perspective of their own position. The 10 groups of this type had a mean age of 21.9 (*SD* = 2.3), a mean number of years of playing experience of 12.8 (*SD* = 1.7) and a mean fielding percentage of 0.91. On average, each group has been playing together as teammates for 1.1 (*SD* = 0.5) years at the time of the study.

*Non-teammates –* four players who currently did not play on the same team together and were asked to make judgments from the viewing perspective of their own position. The 10 groups of this type had a mean age of 20.9 (*SD* = 2.0), a mean number of years of playing experience of 11.6 (*SD* = 2.0) and a mean fielding percentage of 0.93. These groups were formed randomly with the only requirements being that all members currently played for a Division 1 NJCAA team and that all four infield position were represented in each group.

*Scrambled Teammates* – four players who currently played on the same team together and were asked to make judgments from the viewing perspective different from their own position. The 10 groups of this type had a mean age of 22.0 (*SD* = 1.9), a mean number of years of playing experience of 12.9 (*SD* = 2.1) and a mean fielding percentage of 0.92. On average, each group has been playing together as teammates for 1.2 (*SD* = 0.5) years at the time of the study.

One-way ANOVAs revealed that there were no significant differences in age, years of playing experience, or fielding percentage for the three groups, *p*’s all >0.5, all $\eta_{p}^{2}$ ’s all <0.1.

### Apparatus

**FIGURE 2:**
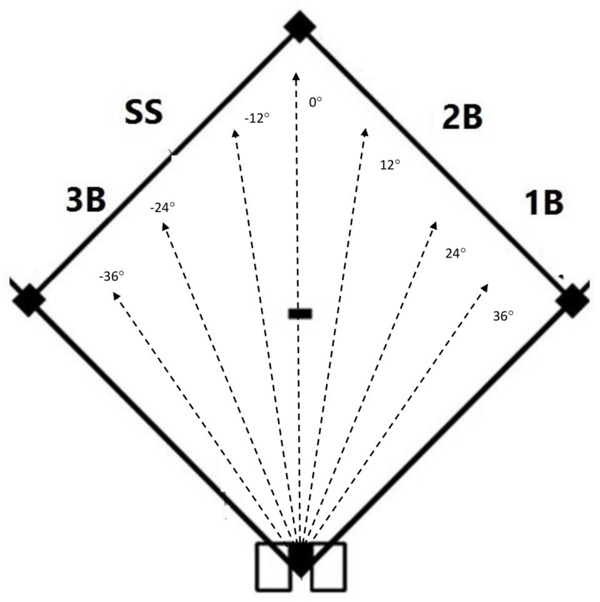
Illustration of the hit trajectories used in the videos. On each trial, a ball was launched onto the ground from home plate (black diamond at bottom of figure) at one of 7 possible angles (dashed lines) with an angle of 0° being a ball hit directly straight ahead over second base (black diamond at top of figure). The 1B (first baseman), 2B (second baseman), SS (shortstop), and 3B (third baseman) show the approximate positions of the four cameras.

Each participant viewed HD videos presented on a 61 cm (24″) Dell Ultra monitor (resolution 1024 × 768) of standard baseballs being projected from a ball launching machine (Sports Tutor ProLite^TM^). Participants watched the videos while seated from a viewing distance of 57 cm. No chin rest was used. Balls were projected at a speed of 11 m/s (25 mph) onto the ground of 15 m (50 ft) side-length, practice baseball diamond. As illustrated in **Figure 2**, there were 7 different lateral launch angles (-36, -24, -12, 0, 12, 24, and 36°), where -45° was the left field line, 0° was over second base and 45° was the right field line. Balls were filmed simultaneously using four Go Pro Hero 4 cameras with 1080p resolution and 60 frames per second. Only the ball and the field were shown in the videos (i.e., the other cameras and players were not visible). The cameras were mounted on tripods and placed in the standard positions of the four infielders. Specifically, each camera was placed 3 m (10 ft) behind and 3 m to the side of the base. The camera height was 1 m, a value chosen to represent the eye height of an average infielder when they are in the “ready position” (i.e., knees and back bent, glove at knee level). **Table 1** shows the approximate launch angles from each of the four infielder/camera locations. Videos were edited so that the view of the ball was occluded and replaced with a mask (a pattern of random black and white dots) after 250 ms. The mask remained on the screen until the player made a response after which the screen was blanked. The inter-trial interval was 500 ms. The viewing duration was chosen based on previous research using temporal occlusion to investigate the return of a tennis serve (e.g., Farrow et al., 2005) which has employed viewing windows of 200–300 ms. To our knowledge, there is no previous research which has used temporal occlusion to study baseball fielding. In the arrangement used in the present study, a ball launched directly at the camera would reach the camera location in roughly 1.6 s for the first and third base locations and 1.9 s for the shortstop and second base locations. Therefore, the 250 ms viewing duration represented approximately 15% of the total flight time for the first and third base positions and 13% of the flight time for the shortstop and second base positions.

**Table 1:** Approximate ball trajectory angles (in degrees) from each viewing position.

Home	1B	2B	SS	3B
-36	-66	-54	-18	-6
-24	-54	-42	-6	6
-12	-42	-30	6	18
0	-30	-18	18	30
12	-18	-6	30	42
24	-6	6	42	54
36	6	18	54	66

### Procedure

Participants were instructed that their task was to verbally indicate as quickly and accurately as possible what they would do for each ground ball. Participants were given two response choices: say “ball” if they would attempt to field the ball, or say the name of the base they would cover if they decided they would let another player on the infield field it. They were further instructed to assume that the other infielders were in their “standard positions in a situation in which the bases were loaded and there were 0 outs.” Responses were recorded using an Audio-Technica PRO 8HEcW headset microphone and audio files were analyzed using a PsychoPy to determine reaction time and ensure synchronization between the videos and audio recordings. After completing 5 practice trials, all participants completed 70 experimental trials representing 10 presentations of the 7 different trajectories presented in random order. Participants were told that they would be performing the experiment simultaneously (in different rooms) with the other three players in their group who would be viewing the videos from different angles. The four participants viewed the videos on separate monitors and could not hear the responses made by the other participants. At no time did participants receive feedback about their responses. For the teammates and non-teammates groups, each player viewed the video from the camera corresponding to their own position. With reference to **Figure 2**, for the scrambled teammates group, the first baseman (1B) viewed the video shot from the shortstop’s (SS) perspective (and vice versa) and the second baseman (2B) viewed the video shot from the third baseman’s (3B) perspective (and vice versa). Each group of four participants waited together (and were free to converse with each other) for 15 min before the study began. They were not told about the specifics of the experiment until they were in separate rooms and were not given a chance to talk to each other again until the experiment was completed.

### Data Analysis

Two different dependent measures were used: coordination score and decision time. Coordination score was a measure of the combined effectiveness of the responses made by the group of four players. To calculate this, we first had two experienced NJCAA baseball coaches watch the videos of the 7 ball trajectories (with no occlusion) and indicate which infielder they felt should attempt to field the ball assuming equal skills among all teammates. These assessments were highly consistent with the coaches producing the same response for all 7 trajectories^1^. The coaches’ choices were then used to assess the group response on each trial using the following scoring system:

*3 points*: Coaches’ choice player goes for the ball, all bags covered by other players in the group.
*2 points*: Player other than coaches’ choice goes for the ball, all bags covered by other players in group.
*1 point*: Two or more players in group indicate they would get the ball (therefore, not all bags covered).
*0 point*: No players in the group indicate they would get the ball.

Mean coordination scores were then calculated for each trajectory by averaging the score for the 10 repeats. Mean decision times were calculated for each trajectory by averaging the times for the four participants then averaging across the 10 repeats. These variables were then analyzed using separate 3 × 7 mixed ANOVAs with group (teammates, non-teammates and scrambled teammates) as a between subjects factors and launch angle as the within subjects factor.

## Results

**FIGURE 3:**
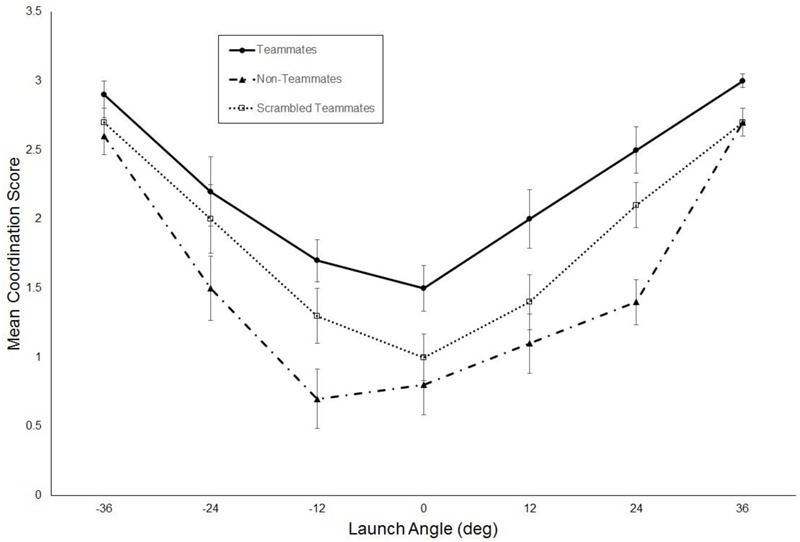
Mean coordination scores plotted as a function of ball launch angle. Error bars are standard errors.

**Figure 3** shows the mean coordination score for the three groups plotted as a function of launch angle. The ANOVA performed on these data revealed a significant main effect of group, *F*(2,27) = 23.8, *p* < 0.001, $\eta_{p}^{2}$ = 0.64. Independent samples *t*-tests (with Bonferroni correction, *p* = 0.017) revealed that the coordination score was significantly higher for the teammates group as compared to both the non-teammates, *t*(18) = 7.4, *p* < 0.001, *d* = 3.3, and scrambled teammates, *t*(18) = 3.9, *p* = 0.001, *d* = 1.8, groups. Furthermore, the coordination score for the scrambled teammates group was significantly higher than for the non-teammates, *t*(18) = 2.9, *p* = 0.009, *d* = 1.3. There was also a significant main effect of launch angle, *F*(6,162) = 28.9, *p* < 0.001, $\eta_{p}^{2}$ = 0.52. As can be seen in **Figure 3**, this occurred because coordination scores were higher for balls launched closer to the foul lines (i.e., larger angle) as opposed to those traveling up the middle. The group × launch angle was not significant, *p =* 0.77, $\eta_{p}^{2}$ = 0.05.

**FIGURE 4:**
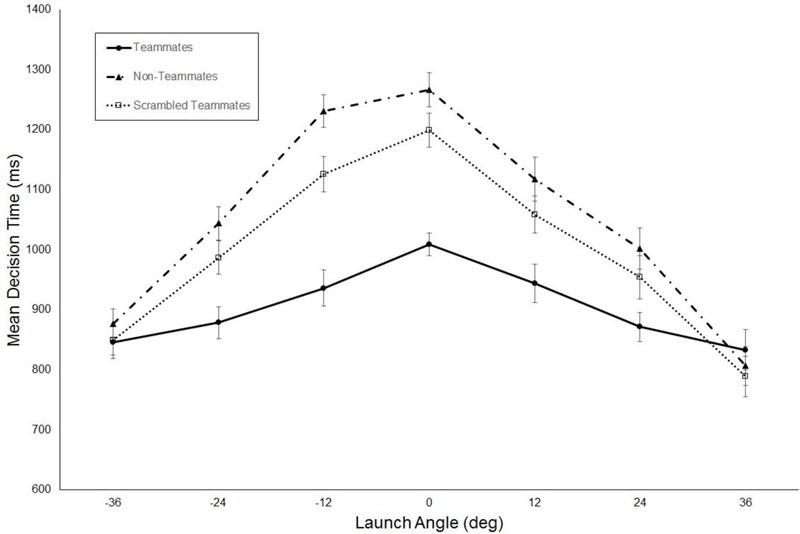
Mean decision times plotted as a function of ball launch angle. Error bars are standard errors.

**Figure 4** shows the mean decision times for the three groups plotted as a function of launch angle. The ANOVA performed on these data revealed significant main effects of group, *F*(2,27) = 54.7, *p* < 0.001, $\eta_{p}^{2}$ = 0.8, and launch angle, *F*(6,162) = 47.4, *p* < 0.001, $\eta_{p}^{2}$ = 0.63. However, these effects were qualified by a significant group × launch angle interaction, *F*(12,162) = 3.6, *p* < 0.001, $\eta_{p}^{2}$ = 0.21. As apparent in **Figure 4**, this interaction occurred because the differences in decision times between groups occurred for the smaller launch angles. Independent samples *t*-tests (with Bonferroni correction, *p* = 0.006) revealed that the decision time was significantly shorter for the teammates group as compared to the non-teammates for the 0° [*t*(18) = 7.0, *p* < 0.001, *d* = 2.8] 12° [*t*(18) = 4.3, *p* < 0.001, *d* = 5.6] and -12° [*t*(18) = 7.0, *p* < 0.001, *d* = 4.8] launch angles. Similarly, the mean decision times were significantly shorter for the teammates group as compared to scrambled teammates group for the 0° [*t*(18) = 4.7, *p* < 0.001, *d* = 3.8], 12° [*t*(18) = 3.1, *p* = 0.003, *d* = 1.7], and -12° [*t*(18) = 3.1, *p* = 0.003, *d* = 3.7], launch angles. The decision time for the scrambled teammates group was significantly shorter than for the non-teammates for the 12° launch angle, *t*(18) = 3.8, *p* = 0.001, *d* = 3.3.

## Discussion

A novel, joint decision making paradigm was used to assess team cognition in baseball infielders. Because it is assumed that team coordination is enhanced through experience performing together, a first requirement of any new methodology is that it shows sensitivity to team experience. This was indeed the case in the present study as players who currently played on the same team (the teammates group) made more coordinated decisions about how to react to a hit ball than players from different teams (the non-teammates group). For balls hit near the middle of the field, the teammates group also made significantly faster decisions. The differences between these groups suggest that coordination in this situation is not achieved through a generic knowledge of how to play a particular position. Instead, we propose that it is due to the fact that the teammates possess both knowledge about how their teammates will respond to particular game situations and knowledge about their teammates action capabilities (e.g., their lateral speed or “range”). This appears to be knowledge that a randomly grouped selection of non-teammates does not have. It is also important to note that our paradigm eliminated some possible explanations for why this effect might be seen if only player movements were examined. Specifically, our joint occlusion paradigm removed the ability to use any verbal or non-verbal communication and prevented players from seeing how their teammates reacted before making their own decision.

An unexpected finding of the present study, that was inconsistent with our second hypothesis, was that teammates “playing out of position” (the scrambled teammates group), made quicker (for balls with small launch angles) and more coordinated decisions than non-teammates viewing the videos from their typical playing position. This suggests that knowledge about the action capabilities of one’s teammates is more important for team coordination than knowledge about how to play one’s position at an individual level. These results are consistent with the idea that joint action in sport involves perceiving both one’s own affordances for action and those of one’s teammates (Fajen et al., 2008).

This team-based occlusion paradigm successfully distinguished three team configurations and different levels of game difficulty. Within the baseball infielder context used in present study, there are several interesting questions that could be addressed with this paradigm in future studies. First, the occlusion time could be systematically manipulated to investigate when decisions are made as has been used for individual decisions in sport (e.g., Abernethy and Russell, 1984). Second, the camera/player positions could be varied from trial to trial to determine how players take into account their relative starting positions in making their decisions. Finally, it would be interesting to use this team-based occlusion method to assess how players respond to the infield shifts (e.g., moving the first baseman, second baseman and shortstop all on the right side of the infield) that are becoming increasingly common in baseball (Los Angeles Times, 2015). Note, the scrambled teammates condition used in the present study was purposely designed to be different than the typical shifts made by infielders. Future work should also extend this paradigm to other team domains in which rapid decisions are needed for coordinated action (e.g., fire-fighting, special forces, and paramedics). Finally, other team configurations can be tested, as well as interventions predicted to improve team coordination (e.g., coaching, simulation training).

The methodology used in the present study deliberately simplified the task of making decisions in a baseball infield by restricting the information available to players to only the flight of the ball. It will be important for future research to add in other sources of information that are available in a real game. First, players should be allowed to communicate with each other, both verbally or non-verbally. Yelling “I got it” or “mine” is an essential part of baseball that players are taught from an early age (Delmonico, 1996). Furthermore, non-verbal communication (e.g., pointing or waving) is also commonly used in baseball (Delmonico, 1996) and has been shown to be critical for team coordination in other sports (e.g., LeCouteur and Feo, 2011). A second limitation of the current paradigm that should be addressed in the future is that the views seen by the players were static and were not yoked to their own head and body movements. This is important because actively exploring the environment can create additional perceptual information (e.g., head movements provide motion parallax information about the relative depth of objects) and experienced performers do seem to use this general strategy (e.g., Huet et al., 2011). One possible way of adding both of these information sources would be to use a virtual reality simulation of infield scenarios in which each a player’s view is yoked to their head movement and players can communicate via a headsets like in a multiplayer video game.

## Author Contributions

All authors listed, have made substantial, direct and intellectual contribution to the work, and approved it for publication.

## Conflict of Interest Statement

The authors declare that the research was conducted in the absence of any commercial or financial relationships that could be construed as a potential conflict of interest.

## Footnotes

1 The choice of which position should field the ball as judged by the coaches was identical to what would be derived by looking at which player was closest to the ball and considering typical player instruction. As shown in **Table 1**, for the -36, -12, 12 and 36° trajectories there was one fielder that had the smallest angular separation to the ball. In each of these situations, the coaches’ choices matched the position with the smallest angle. For the -24, 0 and 24° angles there were two players that were equidistant from the ball. The most common throw made by baseball infielders is to first base (1B). Thus, players are typically taught that in the situation where there are two infielders that could field the ball, the player whose momentum is going toward first base should field the ball. In terms of the values shown in **Table 1**, this means when both players have an equal angular separation to the ball, the player with the positive angle should field it. This again exactly matched the choices made by the coaches for the 24, 0 and -24° angles.
